# Investigation of the Ecological Link between Recurrent Microbial Human Gut Communities and Physical Activity

**DOI:** 10.1128/spectrum.00420-22

**Published:** 2022-04-04

**Authors:** Chiara Tarracchini, Federico Fontana, Gabriele Andrea Lugli, Leonardo Mancabelli, Giulia Alessandri, Francesca Turroni, Marco Ventura, Christian Milani

**Affiliations:** a Laboratory of Probiogenomics, Department of Chemistry, Life Sciences, and Environmental Sustainability, University of Parma, Parma, Italy; b GenProbio Srl, Parma, Italy; c Microbiome Research Hub, University of Parma, Parma, Italy; University of California, San Diego

**Keywords:** athletes, sedentary, microbiota, metagenomics, meta-analysis

## Abstract

Emerging evidence has shown an association between the composition of intestinal microbial communities and host physical activity, suggesting that modifications of the gut microbiota composition may support training, performance, and post-exercise recovery of the host. Nevertheless, investigation of differences in the gut microbiota between athletes and individuals with reduced physical activity is still lacking. In this study, we performed a meta-analysis of 207 publicly available shotgun metagenomics sequencing data of fecal samples from athletes and healthy non-athletes. Accordingly, analysis of species-level fecal microbial profiles revealed three recurring compositional patterns, named HPC1 to 3, that characterize the host based on their commitment to physical activity. Interestingly, the gut microbiome of athletes showed a higher abundance of anti-inflammatory, health-promoting bacteria than that of non-athletic individuals. Moreover, the bacterial species profiled in the gut of professional athletes are short-fatty acid producers, which potentially improve energy production, and therefore sports performances. Intriguingly, microbial interaction network analyses suggested that exercise-induced microbiota adaptation involves the whole microbial community structure, resulting in a complex microbe-microbe interplay driven by positive relationships among the predicted butyrate-producing community members.

**IMPORTANCE** Through metagenomic analyses, this work revealed that athletes have a gut-associated microbial community enriched in butyrate-producing species compared with non-athletes. This evidence can support the existence of a two-way association between the host’s lifestyle and the gut microbiota composition, with potential intriguing athletic performance outcomes.

## INTRODUCTION

The human gut harbors a complex community of microorganisms, commonly referred to as human gut microbiota, which is well-known to play a role in nutrient uptake, vitamin synthesis, energy harvest, inflammatory modulation, and host immune response (1–3). In turn, numerous host-dependent factors, such as genetics, age, antibiotic use, and diet, can affect the gut microbiota resulting in a highly dynamic and individual gut ecosystem (4). Recently, it has been argued that physical activity can influence gut microbiota composition, depending on the type, intensity, and exercise duration. The gut microbiota, in return, may affect the athlete’s health and performance (5). Indeed, if moderate exercises (50% to about 70% of the maximum heart rate) (6) have been reported to increase the overall gut microbiota’s (bio)diversity (7), prolonged endurance exercises (70% to about 85% of the maximum heart rate) (6) have been linked with an increased abundance of gut bacterial species producing short-chain fatty acids (SCFAs) (8). In particular, members of the *Veillonella* genus, along with the metabolic pathways that this taxon utilizes for lactate conversion to propionate, have been detected with elevated abundances in athletes (9), thereby contributing to host metabolic efficiency by increasing energy availability, and thus ultimately influencing athlete performance (10). Moreover, a recent study involving professional and competitive unprofessional cyclists showed that a high training load of the cyclists corresponds to a high abundance of gut-associated *Prevotella* genus members (11). Notably, the presence of this genus has been correlated with increased metabolism of branch chain amino acids, i.e., leucine, valine, and isoleucine (11), which stimulates muscle protein synthesis and accelerates recovery (12). Furthermore, athletes generally consume higher energy diets than sedentary individuals, maintaining a high consumption of carbohydrates and proteins and a low-fat intake, with implications in gut microbiota composition (13).

In this context, our study aimed to explore the microbial communities inhabiting the gut of athletes and non-athletic individuals to highlight compositional and structural differences at the species level. For this purpose, we performed a meta-analysis employing 207 shotgun metagenomics data sets retrieved from public repositories.

## RESULTS AND DISCUSSION

### Meta-analysis of athletic and non-athletic individuals: data set selection and bioinformatics.

Public repositories were screened for all available shotgun metagenomic data sets of the gut microbiomes of the athletes and non-athletic individuals. Specifically, we selected fecal metagenomics data from multiple sources to avoid the limitations of a single-center study. Nevertheless, combining existing data from different studies could lead to biased results due to the different strategies used to generate data sets. In particular, while the DNA extraction method has been shown to produce a little impact on the microbial structure of samples with high microbial load (14), the diverse sequencing protocols could produce different results due to differences in sequence read length and different methodologies exploited to determine the nucleotide sequences. Accordingly, to achieve high resolution of the input data and avoid the above-mentioned bias, we focused only on metagenomic data sets obtained by Illumina sequencing platform.

In detail, shotgun metagenomic sequencing data of 207 fecal samples from 107 non-athletes and 100 athletes engaged in different types of sport (cyclist, rugby players, rower, runner, and marathon athletes) were collected from six different studies (9, 11, 15–18) and submitted to a meta-analysis aimed at elucidating the microbial species composition (Table S1). After quality filtering and removal of reads mapping against the Homo sapiens genome, we obtained a collection of high-quality metagenomic samples with an average of 11,700,594 reads per sample (Table S1).

As previously suggested (7), the evaluation of the alpha-diversity, expressed as the species richness, showed statistically significant differences between the gut microbiomes of non-athletic individuals and athletes, with this latter showing a higher intestinal microbial biodiversity (average of 30 versus 34 species with relative abundance > 0.05%, *t* test *P*-value < 0.05) (Table S2). Similarly, analysis of inter-individual variability through PCoA revealed statistically significant differences in the composition of fecal microbiota between athletes and non-athletes (PERMANOVA *P*-values < 0.05) regardless of ethnic-geographic location, gender, sport type, and study cohort (PERMANOVA *P*-values > 0.05), reflecting the notion that exercise and exercise-related factors can shape the human gut microbial communities (Fig. S1a).

### Taxonomic-based sample clustering and identification of the high prevalence clusters.

**FIG 1:**
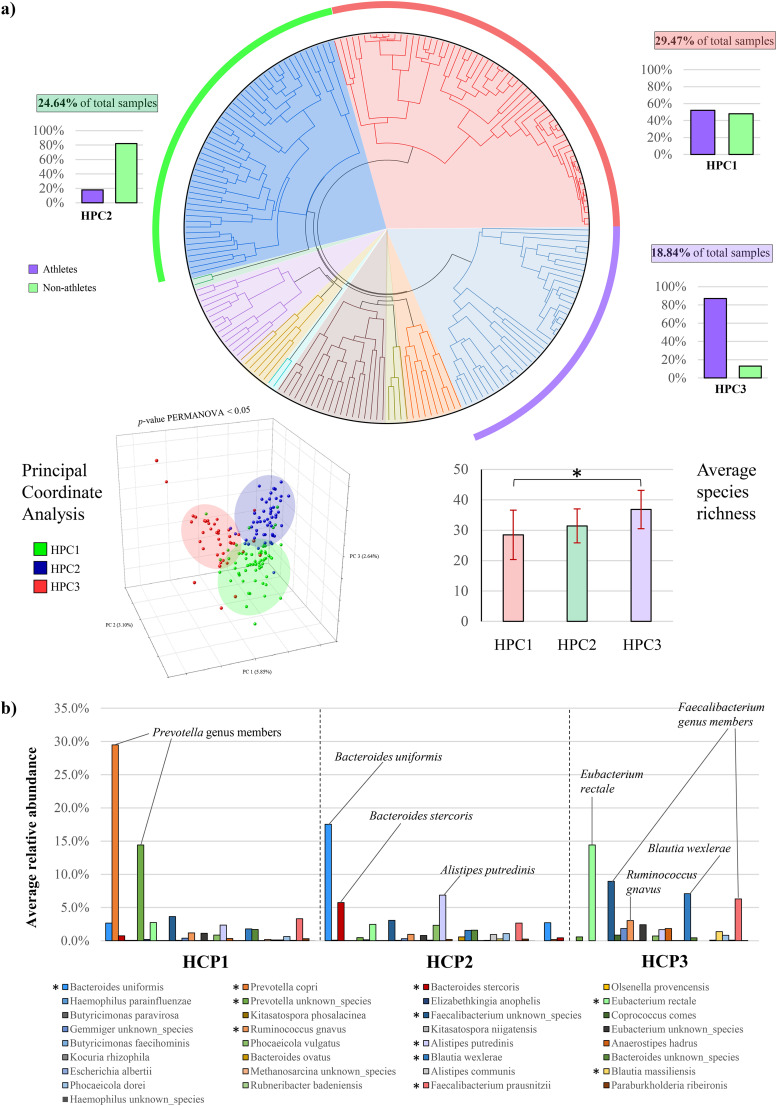
Cluster analysis of the 100 athletes and 107 non-athletes subjects based on gut-associated microbial community composition. Panel a shows the circular HCL-based dendrogram resulting from metagenomic sample clustering that led to the definition of the three high prevalence cluster (HPCs). The proportions of metagenomic samples from athlete and non-athletic individuals in each HPC are reported through histograms outside the circle. Below, alpha- and beta-diversity analyses involving the three HPCs are depicted through a PCoA plot and a bar chart, respectively. In panel b, the microbial taxonomic composition is visualized through a bar chart showing the average relative abundance of each taxon at the species level. The main bacterial species showing statistically significant differences between HPCs are highlighted with asterisks on the chart legend.

Hierarchical clustering (HCL) analysis was performed in combination with the Silhouette method (9), employing the species-level relative abundance data to capture recurrent different taxonomic profiles from metagenomic samples. This approach led to obtaining a statistically optimal number of 10 sample clusters based on their different bacterial composition, representing the community state types (CSTs), i.e., the recurring microbial patterns observed across the investigated cohort of individuals (Fig. 1a, Fig. S1b). Among these, three were identified as the most recurrent microbial profiles, referred to as high prevalence clusters (HPCs), covering individually at least 15% of the samples and collectively 73% of the subjects included in the meta-analysis (Fig. 1a, Table S3).

Integration of the HCL analysis with the available metadata highlighted peculiar associations between HPCs and physical activity levels. In detail, while HPC1 showed a mixed composition (52% of athletes and 48% of control individuals), HPC2 encompassed 82% of non-athletes and HPC3 included 87% of athletes (Fig. 1a, Table S3). To note, after accounting for the study of origin, only 5% of the observed inter-samples variability was explained, demonstrating that geographic location and sample processing methods do not significantly impact on the microbial composition of the subjects included in HPC3 (Fig. S1c). Consistently, the subjects included in the above-mentioned HPCs showed statistically diverse gut microbiome composition, as evidenced by the principal coordinate analysis (PCoA) based on the microbial profiling data at the species level (Fig. 1a). Moreover, microbial biodiversity appears significantly higher in HPC3 (87% of athletes) compared with HPC1 (75% of non-athletes) (average species-richness of 36.8 versus 31.4) (Fig. 1a). As a result, at first glance, it seems that the gut microbiota of athletes is significantly diverse and more complex in terms of taxonomic composition compared to those of subjects with a more sedentary lifestyle.

In particular, through the use of a polynomial linear model, which allows assessing the variability explained by each species (indicated as Adj. R-Square), we highlighted 33 taxa with a value greater than 0.15 (19), thus representing the bacterial species having the most impact in defining HPC structures (Table S3). In detail, these high-impact taxa covered from 45.86% to 68.80% of the three HPC bacterial compositions and highlighted clear connections between specific taxonomic patterns and the host’s physical activity level, as discussed below (Table S3).

### Dissection of the key microbial players of the gut microbiome of athletes and non-athletic individuals.

In order to catch the association between physical activity and specific taxa, we focused on the 33 microbial taxa individuated above as responsible for the main compositional differences between the three HPCs.

In particular, HPC1, composed of 52% of athletes and 48% of non-athletes, was distinct in having high relative abundances of *Prevotella* genus members (average relative abundance of 43.9%) (Fig. 1b, Table S3), which are considered a common commensal microorganism often associated with high dietary fiber intakes (20).

In contrast, HPC2, composed of 82% of samples from non-athletic individuals, was defined by the presence of *Bacteroides* members, including Bacteroides uniformis with an average relative abundance of 17.5% (Fig. 1b, Table S3), as expected from healthy subjects (21). Indeed, the *Bacteroides* taxon is well-known to represent a large portion of the dominant healthy human gut microbiota, previously reported to characterize one of the three renowned human enterotypes (22). Nevertheless, based on HPC2 composition, a non-athletic lifestyle was associated with increased Alistipes putredinis abundance (average relative abundance of 5.9%) compared with individuals with high physical activity, i.e., HPC3 (Fig. 1b, Table S3). This taxon is a member of a relatively recent genus taxonomically closely related to the *Bacteroidetes* phylum (23), whose role in the gut ecosystem is controversial (24). However, previous studies have suggested an association between *Alistipes* and inflammation and disease, including cardiovascular disease and colorectal cancer (25, 26).

Of note, HPC3, composed for the 87% of athletes, is characterized by members of *Faecalibacterium* genus, along with Eubacterium rectale and Blautia wexlerae, with average relative abundances of 15.2%, 14.4%, and 7.1%, respectively, thus resulting significantly higher than those of non-athletic individuals (*P*-values < 0.05) (Fig. 1b, Table S3). Interestingly, *F. prausnitzii*, E. rectale, and members of the *Blautia* genus have been linked with beneficial effects in various clinical conditions, including inflammatory bowel diseases, metabolic syndromes, and colorectal cancer (27–29). Moreover, these taxa have been reported to be responsible for butyrate production (30–32), contributing not only to intestinal anti-inflammatory effects but also to host energy metabolism through *de novo* synthesis of glucose and lipids, which are primary sources of energy for the host organism (33, 34).

Remarkably, these findings revealed clear structural differences between the gut microbiota of the athletes and that of subjects with no physical activity, suggesting the importance of athlete gut-associated microorganisms both as supporters of the gut homeostasis as well as a source of compounds that can increase energy harvest, thus possibly improving athlete performances. However, the limited availability of precise information regarding the individual nutrition regimen did not allow further investigation of the correlation between diet and gut microbiota composition. Thus, future studies will need to collect as a wide range of metadata as possible, including dietetic regimes, that could be essential to understanding how exercise and exercise-associated factors affect the gut microbiota-host interactions in athletes.

### Analysis of the interaction networks sustaining the gut microbial community of athletes and non-athletes.

**FIG 2:**
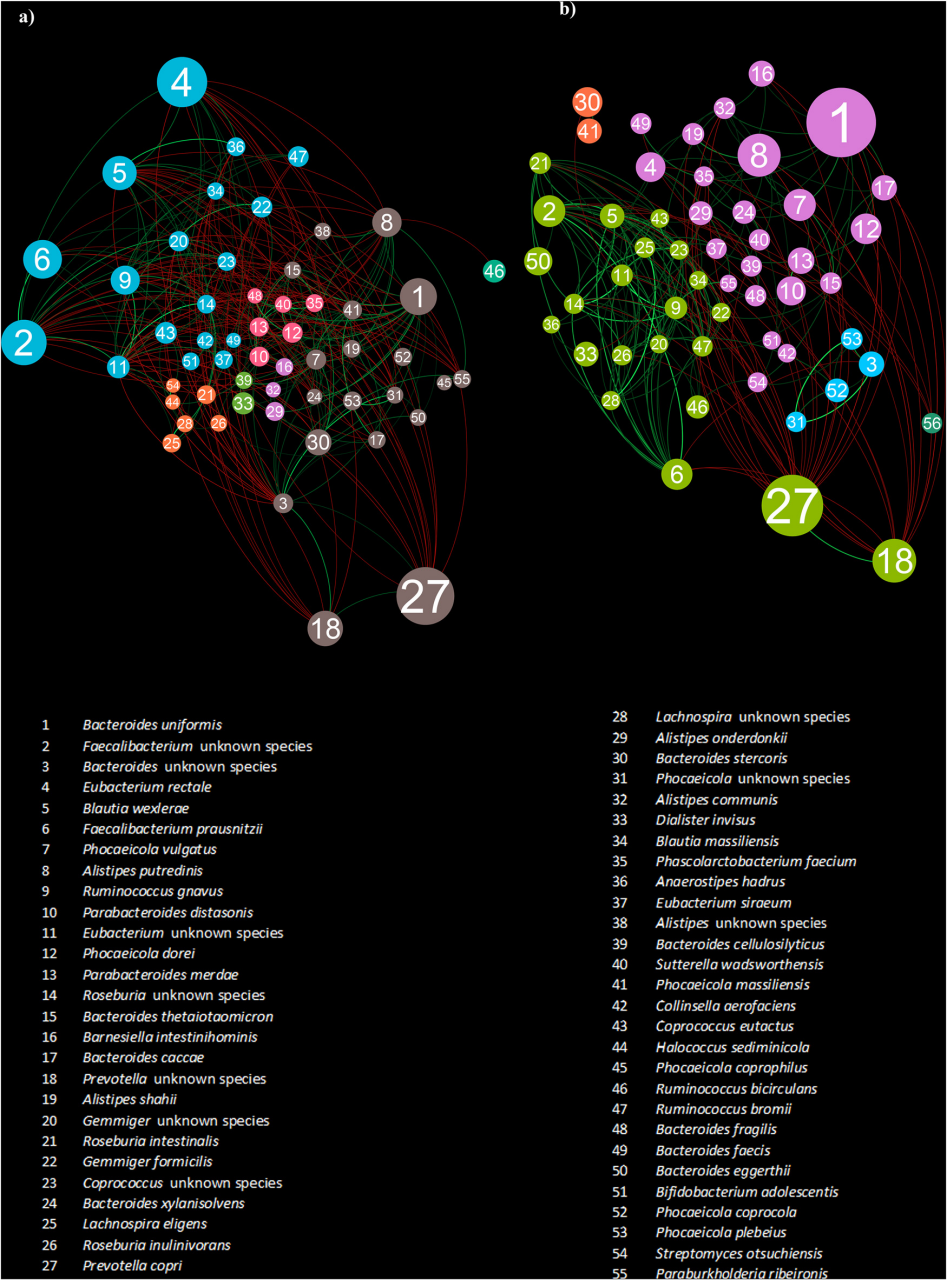
Interaction network supporting the structure of the gut microbial consortia in athletes and non-athletes. Panel a reports the interaction network of athlete gut microbiota, and panel b depicts the interaction network of the fecal microbial community of non-athletic individuals. In the force-driven networks, nodes represent bacterial taxa, and covariance values were used to construct the edges. Red edges correspond to negative correlations, while green edges represent positive associations. The node size is proportional to the relative average abundance of each taxon.

**FIG 3:**
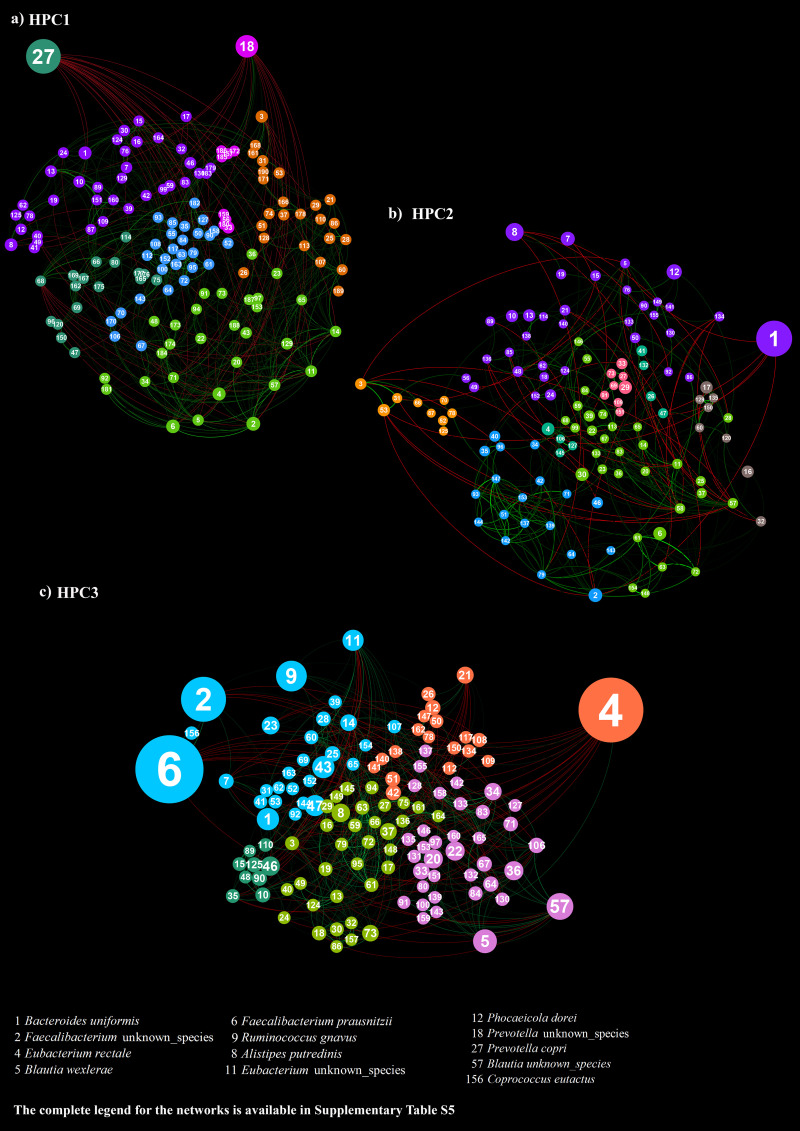
Co-occurrence network characterizing the three HPCs. The networks visualize the covariance relationships between the microbial taxa composing HPC1 (panel a), HPC2 (panel b), and HPC3 (panel c). HPC1 encompasses 52% of athletes and 48% of non-athletic subjects, HPC2 contains 82% of non-athletes, and HPC3 contains 87% of athletes. The complete one-to-one correspondence between node labels and microbial taxa is available in Table S5.

In order to explore the intricate interaction network of the multispecies community constituting the three HPCs, we performed a microbial co-occurrence analysis aimed at highlighting the degree of displacement (negative links) or coexistence (positive links) between species (Table S4). Correlation data were represented by a network of nodes (microbial species) linked in pairs by green edges when the relationships were positive or red edges when they were negative. Furthermore, modularity clusters (MCs) analysis allowed to detect community (sub)structures in networks, i.e., groups of taxa highly interconnected (Fig. 2, Fig. 3). Interestingly, the comparison between the network describing the gut-associated microbial community from athletes and non-athletes revealed a marked difference in the number of statistically significant interactions among taxa (positive and negative links) (Fig. 2). In particular, the microbial network of athletes showed 328 statistically significant associations, of which 62% were positive, in contrast to a total of 223 found gut microbiota members of non-athletic individuals (Table S4). Generally, compared with relatively simple networks, complex interconnected networks have a higher nutritional interaction among community members, such as cross-feeding of essential small molecules, resulting in a more stable microbial consortium with improved resilience to ecosystem disturbances (35). In addition, among the taxa with a prominent role in athlete’s gut microbiota structure, we found species belonging to *Faecalibacterium*, *Eubacterium*, *Ruminococcus*, and *Blautia* genera that are thought to promote intestinal barrier integrity and prevent inflammation (36). Accordingly, these results suggested that the microbial community of athletes exhibits improved stability compared with the gut microbiome of non-athletic individuals, pointing to the importance of microbial synergism among health-promoting species in sustaining the exercise-induced microbiome changes.

### Co-occurrence network analyses of HPCs1 to 3.

Focusing on individual HPC-derived networks, microbial correlation analysis of HPC1, which showed a mixed composition of non-athletic and athletic individuals, it is worth mentioning that members of *Prevotella* genus (node 27 and 18), such as Prevotella copri (node 27), tend to dominate their intestinal ecological niche. In addition, this taxon negatively correlated with other typical key members of the healthy gut-associated microbial communities, including B. uniformis, Ruminococcus gnavus, and members of *Faecalibacterium* genus (Fig. 3a, Table S4). Simultaneously, a dense and intricate network of positive associations between minority players (proportion of 94% of the total network interactions) seems to sustain the microbial community structure of HPC1.

Conversely, the HPC2, which covers mainly non-athletic subjects, appeared to be driven by five related keystone taxa, belonging to *Bacteroides* (nodes 1 and 30), *Phocaeicola* (nodes 12 and 41), and *Alistipes* (node 8) genera (Fig. 3b, Table S4). In particular, these taxa were engaged in negative correlations mainly with potentially anti-inflammatory, butyrate-producing bacteria from the genera *Ruminoccoccus*, *Faecalibacterium*, and *Blautia* (28, 37), thus revealing a possible negative impact of a sedentary or low physical activity lifestyle on health-associated commensal bacteria. However, a small-scale subnetwork (light blue) comprising well-known commensals of the healthy human gut microbiota, such as Bifidobacterium longum, Bifidobacterium adolescentis, and Collinsella aerofaciens (Fig. 3b, Table S4), despite their low relative abundance in non-athletic subjects (<1%), seem to play a pivotal role in establishing positive correlations with other minor microbial players, regulating a large part of the microbial consortium characterizing healthy non-athletic individuals.

Interestingly, interaction networks describing the gut-associated microbial community of athletes, i.e., HPC3 (Fig. 3c, Table S4), showed the highest number of species that, being involved in conspicuous biotic interactions, seem to influence the whole-community dynamics of the athlete gut microbiota. Indeed, as previously mentioned, health-associated species, i.e., Faecalibacterium prausnitzii (node 6), Blautia wexlerae (node 5), and Eubacterium rectale (node 4), along with Ruminococcus gnavus (node 9), act as keystone taxa in HPC3, exerting considerable control on the entire community structure (Fig. 3c, Table S4). In addition, these taxa are involved in strong positive associations (Spearman correlation coefficient value > 0.5) with members of the *Coprococcus* and *Roseburia* genera that, being part of commensal bacteria producing SCFAs, primarily butyrate, exert a positive influence on intestinal barrier maintenance, colonic motility, and anti-inflammatory processes (38–40). Besides, additional low-abundance members appear to have significant effects on the intestinal niche, reflecting the existence of a complex and solid ecosystem. As a result, removing a few species likely does not lead to a dramatic shift in the composition. Taken together, these findings support the notion that exercise can affect the gut microbiota composition, inducing qualitative and quantitative changes that may confer beneficial effects to the host and possibly to athletic performance.

### Conclusion.

Accumulating evidence has suggested a bidirectional association between physical activity and the composition of the microbial communities inhabiting the human intestinal environment (41). Indeed, differences in the gut microbiota composition have been observed between athletes and non-athletes, with this latter showing an increased abundance of short-chain fatty acids (SCFAs)-producing bacterial species (8, 42). In turn, the gut microbiota is thought to play a significant role in amino acid and carbohydrate host metabolism, likely indirectly influencing athlete health, training, sports performance, and post-exercise recovery (41, 43).

In this framework, a metagenomic analysis was performed by exploiting publicly available shotgun metagenomic data sets with the aim to provide insights into the gut-associated microbial community structure in athletes. In particular, a collection of 100 metagenomic samples from athletes and 107 from healthy non-athletic individuals allowed us to identify three high prevalence clusters (HPC1 to 3), i.e., recurring patterns of microbial composition. Interestingly, the gut microbiome of athletes (HPC3) showed higher biodiversity with an increased abundance of gut-associated health-promoting bacterial species compared to non-athletes.

In particular, SCFAs-producing species such as *F. prausnitzii*, E. rectale, B. wexlerae, and *R. gnavus*, were associated with athlete physical activity, revealing their possible contribution to the host health, regulating inflammation and immune system, as well as athlete’s energy acquisition and sport performances. Moreover, an intricate and solid network of biotic interactions sustained by seven health-promoting key species and a range of concurrent low-abundance taxa seems to characterize the microbial community of athletes. In contrast, a less clustered and less inter-connected network was obtained from non-athletic subjects. Based on these findings, it appears that exercise induces gut microbiota changes resulting in an increased abundance of bacteria with potential health benefits, such as SCFAs producers, cooperating in complex, interconnected microbial communities, with possible positive implications on sports performance. Future detailed functional analysis addressing the metabolic capability of the gut microbiota will aid in elucidating the connection between microbial-derived metabolites and athletic versus non-athletic lifestyle.

## MATERIALS AND METHODS

### Metagenomic sample collection.

With the aim to explore the differences in the gut microbiome composition between athletes and non-athletic individuals, we retrieved all the publicly available shotgun metagenomic raw data (fastq) from the National Center of Biotechnology Information (NCBI) Sequence Read Archive (SRA) database. Accordingly, to safeguard consistency and equivalence across metagenomic samples from different studies, we selected only those produced through Illumina sequencing method. As a result, we collected 207 shotgun metagenomics samples from six different studies (PRJEB15388, PRJEB28338, PRJEB32794, PRJNA472785, PRJNA305507, PRJEB20054), of which 100 corresponded to athlete gut microbiomes, and 107 were from healthy non-athletes (Table S1). In addition, the respective metadata regarding health status, training type, exercise intensity level, and diet were also collected (Table S1).

### Metagenomics data processing and taxonomic profiling.

The fastq raw data obtained from publicly repositories were submitted to quality filtering to remove sequence reads with low quality scores (<25). Subsequently, removal of reads mapping on the hg19 human reference genome was performed to exclude host DNA. This process allowed to achieve an average of 11,700,594 ± 9,886,096 reads per sample that were submitted to downstream analyses. The retained reads were subjected to taxonomic classification using METAnnotatorX2 bioinformatics platform (44), which performs MegaBLAST local alignment of reads (45) to the curated non-redundant sequence database of genomes retrieved from NCBI servers.

For each metagenomic sample, taxonomical biodiversity, i.e., species richness, was calculated as the number of gut-associated bacterial taxa whose sequenced reads had a relative abundance greater than 0.5%. Similarities between samples (beta-diversity) were calculated by Bray-Curtis dissimilarity based on species abundance. The range of similarities is calculated between values 0 and 1. PCoA representation of beta-diversity was performed using ORIGIN 2021 (https://www.originlab.com/2021). In the PCoA each dot represented a sample, distributed in tridimensional space according to its own bacterial composition.

The hierarchical clustering (HCL) of samples was achieved employing bacterial composition at the species level and was calculated through TMeV 4.8.1 software using Pearson correlation as a distance metric based on species-level information. The data obtained was represented by a dendrogram.

### Microbial co-occurrence and network analyses.

Covariance analysis involving the 332 bacterial species obtained by taxonomic profiling of the 207 metagenomic fecal samples was realized employing Kendall’s tau rank covariance analysis (46). Using software Gephi (https://gephi.org/), the obtained correlation coefficients were exploited to build a force-driven network, whose nodes represent bacterial species, and edges define their relationships. The node size is related to the number of interactions of a specific microbial taxon, i.e., the node degree, while the edge color shows the type of interaction, i.e., positive (green) or negative (red).

### Statistical analysis.

ORIGIN 2021 (https://www.originlab.com/2021) and SPSS software (www.ibm.com/software/it/analytics/spss/) were used to compute statistical analyses. PERMANOVA analyses were performed using 1,000 permutations to assess p‐values for differences among populations in PCoA analyses. Furthermore, bacterial abundance differences were tested by t‐test analysis.

### Data availability.

Not applicable.

## References

[1] Rowland I, Gibson G, Heinken A, Scott K, Swann J, Thiele I, Tuohy K. 2018. Gut microbiota functions: metabolism of nutrients and other food components. Eur J Nutr 57:1–24. doi:10.1007/s00394-017-1445-8. PMC5847071 28393285

[2] Yoo JY, Groer M, Dutra SVO, Sarkar A, McSkimming DI. 2020. Gut microbiota and immune system interactions. Microorganisms 8:1587–1522. doi:10.3390/microorganisms8101587. 33076307 PMC7602490

[3] Hakansson A, Molin G. 2011. Gut microbiota and inflammation. Nutrients 3:637–682. doi:10.3390/nu3060637. 22254115 PMC3257638

[4] Hasan N, Yang H. 2019. Factors affecting the composition of the gut microbiota, and its modulation. PeerJ 7. doi:10.7717/peerj.7502. PMC6699480 31440436

[5] Hughes RL, Holscher HD. 2021. Fueling gut microbes: a review of the interaction between diet, exercise, and the gut microbiota in athletes. Adv Nutr 12:2190–2215. doi:10.1093/advances/nmab077. 34229348 PMC8634498

[6] Piercy KL, Troiano RP, Ballard RM, Carlson SA, Fulton JE, Galuska DA, George SM, Olson RD. 2018. The physical activity guidelines for Americans. JAMA 320:2020–2028. doi:10.1001/jama.2018.14854. 30418471 PMC9582631

[7] Clarke SF, Murphy EF, O’Sullivan O, Lucey AJ, Humphreys M, Hogan A, Hayes P, O'Reilly M, Jeffery IB, Wood-Martin R, Kerins DM, Quigley E, Ross RP, O’Toole PW, Molloy MG, Falvey E, Shanahan F, Cotter PD. 2014. Exercise and associated dietary extremes impact on gut microbial diversity. Gut 63:1913–1920. doi:10.1136/gutjnl-2013-306541. 25021423

[8] Hughes RL. 2020. A review of the role of the gut microbiome in personalized sports nutrition. Front Nutr 6. doi:10.3389/fnut.2019.00191. PMC6966970 31998739

[9] Scheiman J, Luber JM, Chavkin TA, MacDonald T, Tung A, Pham L-D, Wibowo MC, Wurth RC, Punthambaker S, Tierney BT, Yang Z, Hattab MW, Avila-Pacheco J, Clish CB, Lessard S, Church GM, Kostic AD. 2019. Meta-omics analysis of elite athletes identifies a performance-enhancing microbe that functions via lactate metabolism. Nat Med 25:1104–1109. doi:10.1038/s41591-019-0485-4. 31235964 PMC7368972

[10] Turpin-Nolan SM, Joyner MJ, Febbraio MA. 2019. Can microbes increase exercise performance in athletes? Nat Rev Endocrinol 15:629–630. doi:10.1038/s41574-019-0250-2. 31391569

[11] Petersen LM, Bautista EJ, Nguyen H, Hanson BM, Chen L, Lek SH, Sodergren E, Weinstock GM. 2017. Community characteristics of the gut microbiomes of competitive cyclists. Microbiome 5:98. doi:10.1186/s40168-017-0320-4. 28797298 PMC5553673

[12] Blomstrand E, Eliasson J, Karlsson HKR, Köhnke R. 2006. Branched-chain amino acids activate key enzymes in protein synthesis after physical exercise. The J Nutrition 136:269S–273S. doi:10.1093/jn/136.1.269S. 16365096

[13] Spriet LL. 2019. Performance Nutrition for Athletes. Sports Medicine (Auckland, NZ) 49. https://pubmed.ncbi.nlm.nih.gov/30671901/. 10.1007/s40279-018-1027-9 PMC6445808 30671901

[14] Sui H-y, Weil AA, Nuwagira E, Qadri F, Ryan ET, Mezzari MP, Phipatanakul W, Lai PS. 2020. Impact of DNA extraction method on variation in human and built environment microbial community and functional profiles assessed by shotgun metagenomics sequencing. Front Microbiol 11. doi:10.3389/fmicb.2020.00953. PMC7262970 32528434

[15] Barton W, Penney NC, Cronin O, Garcia-Perez I, Molloy MG, Holmes E, Shanahan F, Cotter PD, O’Sullivan O. 2018. The microbiome of professional athletes differs from that of more sedentary subjects in composition and particularly at the functional metabolic level. Gut 67:625–633. https://pubmed.ncbi.nlm.nih.gov/28360096/. 28360096 10.1136/gutjnl-2016-313627

[16] O’Donovan CM, Connor B, Madigan SM, Cotter PD, O’Sullivan O. 2020. Instances of altered gut microbiomes among Irish cricketers over periods of travel in the lead up to the 2016 World Cup: a sequencing analysis. Travel Medicine and Infectious Dis 35:101553. doi:10.1016/j.tmaid.2020.101553. 31935465

[17] O’Donovan CM, Madigan SM, Garcia-Perez I, Rankin A, O’Sullivan O, Cotter PD. 2020. Distinct microbiome composition and metabolome exists across subgroups of elite Irish athletes. J Sci Med Sport 23:63–68. doi:10.1016/j.jsams.2019.08.290. 31558359

[18] Cronin O, Barton W, Skuse P, Penney NC, Garcia-Perez I, Murphy EF, Woods T, Nugent H, Fanning A, Melgar S, Falvey EC, Holmes E, Cotter PD, O’Sullivan O, Molloy MG, Shanahan F. 2018. A prospective metagenomic and metabolomic analysis of the impact of exercise and/or whey protein supplementation on the gut microbiome of sedentary adults. mSystems 3. doi:10.1128/mSystems.00044-18. PMC5915698 29719871

[19] Kelsey CM, Prescott S, McCulloch JA, Trinchieri G, Valladares TL, Dreisbach C, Alhusen J, Grossmann T. 2021. Gut microbiota composition is associated with newborn functional brain connectivity and behavioral temperament. Brain Behav Immun 91:472–486. doi:10.1016/j.bbi.2020.11.003. 33157257

[20] Kovatcheva-Datchary P, Nilsson A, Akrami R, Lee YS, De Vadder F, Arora T, Hallen A, Martens E, Björck I, Bäckhed F. 2015. Dietary fiber-induced improvement in glucose metabolism is associated with increased abundance of Prevotella. Cell Metab 22:971–982. doi:10.1016/j.cmet.2015.10.001. 26552345

[21] Zafar H, Saier MH. 2021. Gut Bacteroides species in health and disease. Gut Microbes 13:1–20. https://pubmed.ncbi.nlm.nih.gov/33535896/. 10.1080/19490976.2020.1848158 PMC7872030 33535896

[22] Arumugam M, Raes J, Pelletier E, Le Paslier D, Yamada T, Mende DR, Fernandes GR, Tap J, Bruls T, Batto J-M, Bertalan M, Borruel N, Casellas F, Fernandez L, Gautier L, Hansen T, Hattori M, Hayashi T, Kleerebezem M, Kurokawa K, Leclerc M, Levenez F, Manichanh C, Nielsen HB, Nielsen T, Pons N, Poulain J, Qin J, Sicheritz-Ponten T, Tims S, Torrents D, Ugarte E, Zoetendal EG, Wang J, Guarner F, Pedersen O, de Vos WM, Brunak S, Doré J, Weissenbach J, Ehrlich SD, Bork P, MetaHIT Consortium (additional members). 2011. Enterotypes of the human gut microbiome. Nature 473:174–180. doi:10.1038/nature09944. 21508958 PMC3728647

[23] Rautio M, Eerola E, Väisänen-Tunkelrott M-L, Molitoris D, Lawson P, Collins MD, Jousimies-Somer H. 2003. Reclassification of Bacteroides putredinis (Weinberg et al., 1937) in a new genus Alistipes gen. nov., as Alistipes putredinis comb. nov., and description of Alistipes finegoldii sp. nov., from human sources. Syst Appl Microbiol 26:182–188. doi:10.1078/072320203322346029. 12866844

[24] Parker BJ, Wearsch PA, Veloo ACM, Rodriguez-Palacios A. 2020. The genus alistipes: gut bacteria with emerging implications to inflammation, cancer, and mental health. Front Immunol 11:906. doi:10.3389/fimmu.2020.00906. 32582143 PMC7296073

[25] Jie Z, Xia H, Zhong S-L, Feng Q, Li S, Liang S, Zhong H, Liu Z, Gao Y, Zhao H, Zhang D, Su Z, Fang Z, Lan Z, Li J, Xiao L, Li J, Li R, Li X, Li F, Ren H, Huang Y, Peng Y, Li G, Wen B, Dong B, Chen J-Y, Geng Q-S, Zhang Z-W, Yang H, Wang J, Wang J, Zhang X, Madsen L, Brix S, Ning G, Xu X, Liu X, Hou Y, Jia H, He K, Kristiansen K. 2017. The gut microbiome in atherosclerotic cardiovascular disease. Nat Commun 8. doi:10.1038/s41467-017-00900-1. PMC5635030 29018189

[26] Moschen AR, Gerner RR, Wang J, Klepsch V, Adolph TE, Reider SJ, Hackl H, Pfister A, Schilling J, Moser PL, Kempster SL, Swidsinski A, Orth Höller D, Weiss G, Baines JF, Kaser A, Tilg H. 2016. Lipocalin 2 protects from inflammation and tumorigenesis associated with gut microbiota alterations. Cell Host Microbe 19:455–469. doi:10.1016/j.chom.2016.03.007. 27078067

[27] Mukherjee A, Lordan C, Ross RP, Cotter PD. 2020. Gut microbes from the phylogenetically diverse genus Eubacterium and their various contributions to gut health. Gut Microbes 12:1802866. doi:10.1080/19490976.2020.1802866. 32835590 PMC7524325

[28] Liu X, Mao B, Gu J, Wu J, Cui S, Wang G, Zhao J, Zhang H, Chen W. 2021. Blautia-a new functional genus with potential probiotic properties? Gut Microbes 13:1–21. https://pubmed.ncbi.nlm.nih.gov/33525961/. 10.1080/19490976.2021.1875796 PMC7872077 33525961

[29] Ferreira-Halder CV, Faria A. V d S, Andrade SS. 2017. Action and function of Faecalibacterium prausnitzii in health and disease. Best Pract Res Clin Gastroenterol 31:643–648. doi:10.1016/j.bpg.2017.09.011. 29566907

[30] Nilsen M, Madelen Saunders C, Leena Angell I, Arntzen MØ, Lødrup Carlsen KC, Carlsen K-H, Haugen G, Heldal Hagen L, Carlsen MH, Hedlin G, Monceyron Jonassen C, Nordlund B, Maria Rehbinder E, Skjerven HO, Snipen L, Cathrine Staff A, Vettukattil R, Rudi K. 2020. Butyrate levels in the transition from an infant- to an adult-like gut microbiota correlate with bacterial networks associated with Eubacterium rectale and Ruminococcus gnavus. Genes 11:1245–1215. doi:10.3390/genes11111245. 33105702 PMC7690385

[31] Morrison DJ, Preston T. 2016. Formation of short chain fatty acids by the gut microbiota and their impact on human metabolism. Gut Microbes 7:189–200. doi:10.1080/19490976.2015.1134082. 26963409 PMC4939913

[32] Vacca M, Celano G, Calabrese FM, Portincasa P, Gobbetti M, de Angelis M. 2020. The controversial role of human gut lachnospiraceae. Microorganisms [Internet] 8:573. doi:10.3390/microorganisms8040573. 32326636 PMC7232163

[33] den Besten G, van Eunen K, Groen AK, Venema K, Reijngoud DJ, Bakker BM. 2013. The role of short-chain fatty acids in the interplay between diet, gut microbiota, and host energy metabolism. J Lipid Res 54:2325–2340. doi:10.1194/jlr.R036012. 23821742 PMC3735932

[34] den Besten G, Lange K, Havinga R, van Dijk TH, Gerding A, van Eunen K. 2013. Gut-derived short-chain fatty acids are vividly assimilated into host carbohydrates and lipids. American J Physiology Gastrointestinal and Liver Physiology 305. doi:10.1152/ajpgi.00265.2013. 24136789

[35] Wagg C, Schlaeppi K, Banerjee S, Kuramae EE, van der Heijden MGA. 2019. Fungal-bacterial diversity and microbiome complexity predict ecosystem functioning. Nat Commun 10. doi:10.1038/s41467-019-12798-y. PMC6813331 31649246

[36] Lordan C, Thapa D, Ross RP, Cotter PD. 2020. Potential for enriching next-generation health-promoting gut bacteria through prebiotics and other dietary components. Gut Microbes 11:1–20. doi:10.1080/19490976.2019.1613124. 31116628 PMC6973326

[37] Takahashi K, Nishida A, Fujimoto T, Fujii M, Shioya M, Imaeda H, Inatomi O, Bamba S, Andoh A, Sugimoto M. 2016. Reduced abundance of butyrate-producing bacteria species in the fecal microbial community in Crohn’s disease. Digestion 93:59–65. doi:10.1159/000441768. 26789999

[38] Nie K, Ma K, Luo W, Shen Z, Yang Z, Xiao M, Tong T, Yang Y, Wang X. 2021. Roseburia intestinalis: a beneficial gut organism from the discoveries in genus and species. Front Cell Infect Microbiol 11:757718. doi:10.3389/fcimb.2021.757718. 34881193 PMC8647967

[39] Valles-Colomer M, Falony G, Darzi Y, Tigchelaar EF, Wang J, Tito RY, Schiweck C, Kurilshikov A, Joossens M, Wijmenga C, Claes S, Van Oudenhove L, Zhernakova A, Vieira-Silva S, Raes J. 2019. The neuroactive potential of the human gut microbiota in quality of life and depression. Nat Microbiol 4:623–632. doi:10.1038/s41564-018-0337-x. 30718848

[40] Canani RB, di Costanzo M, Leone L, Pedata M, Meli R, Calignano A. 2011. Potential beneficial effects of butyrate in intestinal and extraintestinal diseases. World J Gastroenterol 17:1519–1528. doi:10.3748/wjg.v17.i12.1519. 21472114 PMC3070119

[41] Aya V, Flórez A, Perez L, Ramírez JD. 2021. Association between physical activity and changes in intestinal microbiota composition: A systematic review. PLoS One 16:e0247039. doi:10.1371/journal.pone.0247039. 33630874 PMC7906424

[42] Mohr AE, Jäger R, Carpenter KC, Kerksick CM, Purpura M, Townsend JR, West NP, Black K, Gleeson M, Pyne DB, Wells SD, Arent SM, Kreider RB, Campbell BI, Bannock L, Scheiman J, Wissent CJ, Pane M, Kalman DS, Pugh JN, Ortega-Santos CP, ter Haar JA, Arciero PJ, Antonio J. 2020. The athletic gut microbiota. J Int Soc Sports Nutr 17. doi:10.1186/s12970-020-00353-w. PMC7218537 32398103

[43] Koh A, de Vadder F, Kovatcheva-Datchary P, Bäckhed F. 2016. From dietary fiber to host physiology: short-chain fatty acids as key bacterial metabolites. Cell 165:1332–1345. doi:10.1016/j.cell.2016.05.041. 27259147

[44] Milani C, Lugli GA, Fontana F, Mancabelli L, Alessandri G, Longhi G, Anzalone R, Viappiani A, Turroni F, van Sinderen D, Ventura M. 2021. METAnnotatorX2: a comprehensive tool for deep and shallow metagenomic data set analyses. mSystems 6. doi:10.1128/mSystems.00583-21. PMC8269244 34184911

[45] Chen Y, Ye W, Zhang Y, Xu Y. 2015. High speed BLASTN: an accelerated MegaBLAST search tool. Nucleic Acids Res 43:7762–7768. doi:10.1093/nar/gkv784. 26250111 PMC4652774

[46] Liu X, Ning J, Cheng Y, Huang X, Li R. 2019. A flexible and robust method for assessing conditional association and conditional concordance. Stat Med 38:3656–3668. doi:10.1002/sim.8202. 31074082 PMC7045600

