# Supplementary material for: Investigation of the Ecological Link between Recurrent Microbial Human Gut Communities and Physical Activity
Source: Microbiol Spectr. 2022 Apr 4;10(2):e00420-22. doi: 10.1128/spectrum.00420-22 (PMC9045144; doi:10.1128/spectrum.00420-22)
Supplement: SUPPLEMENTAL FILE 1 — Supplemental material. Download SPECTRUM00420-22_Supp_1_seq7.pdf, PDF file, 0.5 MB [file spectrum00420-22_supp_1_seq7.pdf]

a)

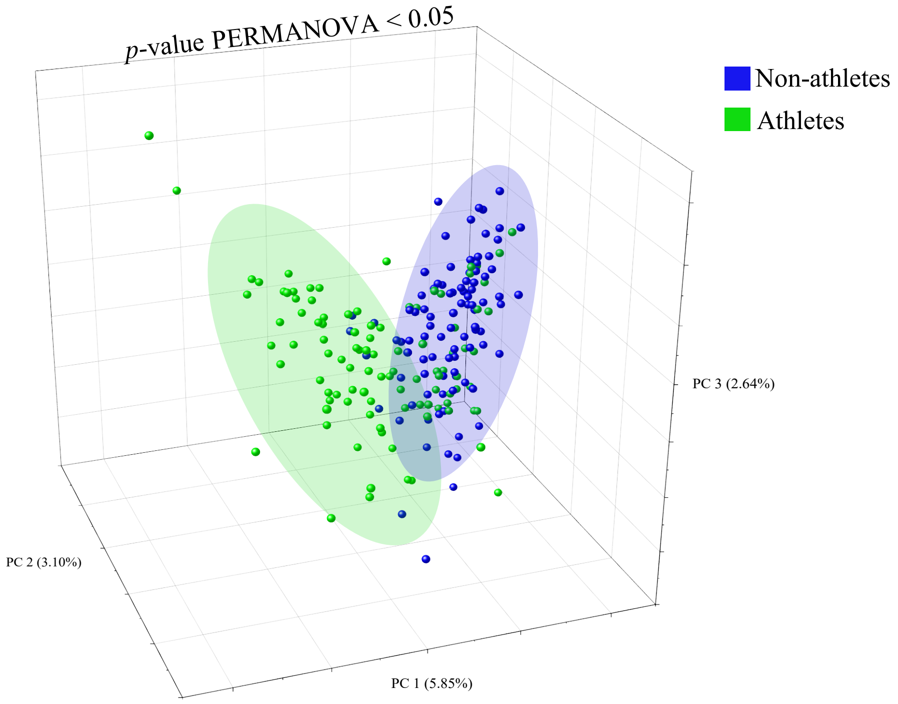

b)

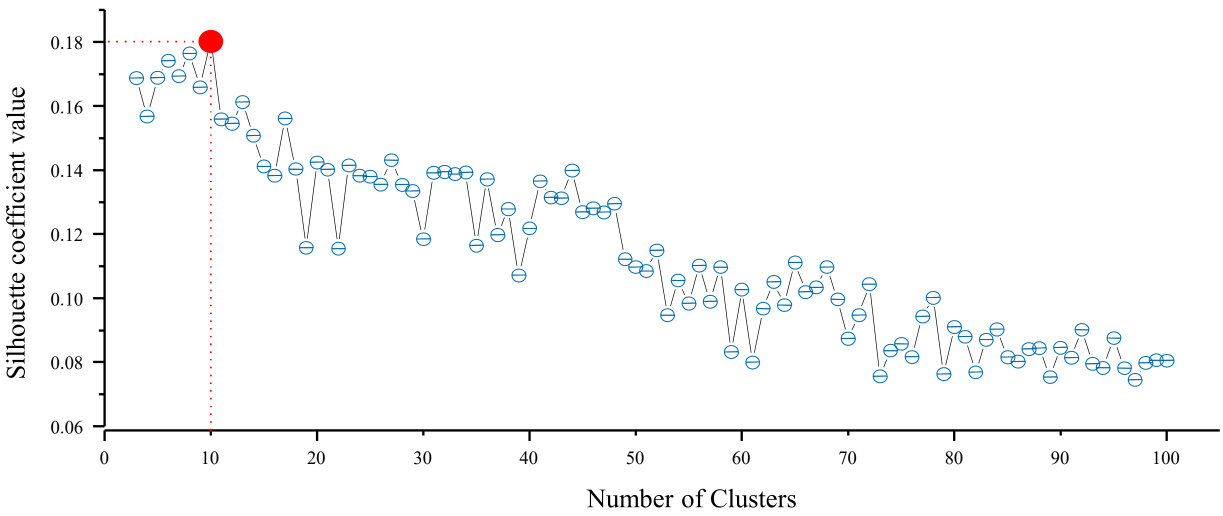

c)

Distribution of the athlete samples of HPC3 based on study of provenance

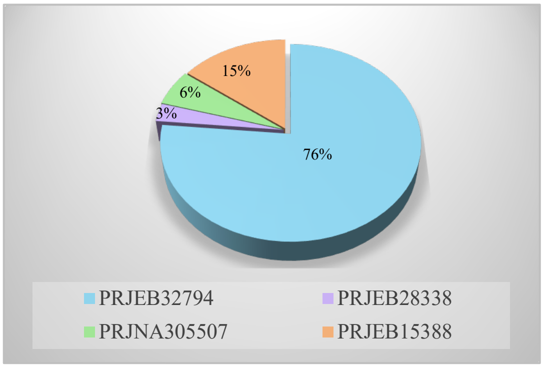

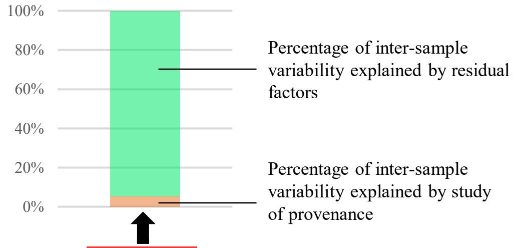

	Df	SumOfSqs	R2	F	Pr(>F)
**Study of provenance**	1	0.226218406	**0.055190907**	1.869276063	0.05
Residual	32	3.872616317	0.944809093	NA	NA
Total	33	4.098834722	1	NA	NA

Figure S1

Figure S1. Principal Coordinate Analysis (PCoA) and Silhouette analyses of the metagenomic datasets of athletes and non-athletic subjects. Panel a displays the plot resulting from PCoA analysis involving the 100 metagenomic samples from athletes and the 107 obtained from non-athletes. Panel c shows the Silhouette analysis used to select the optimal number of sample clusters. The vertical axis reports the Silhouette coefficient values, and the horizontal axis reports the corresponding number of sample clusters. Panel c reports a pie chart describing the composition of HPC3 (87 % athlete samples) according to the studies from which they were retrieved. Corresponding PERMANOVA *p*-value is shown in the side box, and the percentage of the inter-sample variability explained by the study of origin is depicted through a pillar.
